# Deep Learning-Based Multi-Animal Tracking for Behavioral Analysis: From Identity Preservation and Pose Estimation to Social Interaction Inference

**DOI:** 10.3390/ani16203191

**Published:** 2026-10-11

**Authors:** Susumu Takahashi, Hirotsugu Azechi

**Affiliations:** Laboratory of Cognitive and Behavioral Neuroscience, Graduate School of Brain Science, Doshisha University, Kyotanabe 610-0394, Japan

**Keywords:** multi-animal tracking, markerless pose estimation, deep learning, identity preservation, social behavior, behavioral classification, behavioral segmentation, interaction analysis, post-processing, data quality

## Abstract

Deep learning-based video analysis has enabled markerless pose estimation, identity-preserved tracking, and quantitative behavioral analysis in a range of experimental animal systems. However, extending these approaches from single animals to interacting groups introduces persistent challenges, including body-part assignment, identity preservation, occlusion, crowding, missing keypoints, and reduced reliability during close social interactions. This structured narrative review examines multi-animal tracking based primarily on 2D RGB video and its connection to downstream behavioral analysis. Literature was identified by searching PubMed, bioRxiv, arXiv, Scopus, and IEEE Xplore on 15 September 2026, supplemented by citation tracking, and incorporated iteratively according to relevance to the review topics. The review highlights that tracking accuracy alone does not determine whether data are suitable for a behavioral question. Identity errors, missing keypoints, and temporal processing can affect behavioral classification, social metrics, and directed interaction estimates. It also emphasizes that the required tracking representation depends on the intended analysis: identity-preserved trajectories may support movement and group-level measures, whereas full-body pose may be required for posture- or orientation-dependent interactions. Reliable use of multi-animal tracking requires quality control and data preparation matched to the intended behavioral analysis.

## Simple Summary

Deep learning allows computer programs to learn patterns from examples and use them to locate animals and their body parts in video recordings. These tools allow researchers to follow several animals at once and measure their movements and social interactions. However, the task becomes difficult when animals look alike, touch one another, or block one another from view. The software may then lose body positions, confuse one animal with another, or produce measurements that are difficult to trust. These problems often occur during close contact, precisely when important social behaviors take place. This review describes recent methods for following multiple animals and explains how the resulting position and movement measurements can be prepared and used to study behavior. It also considers how tracking errors can affect the recognition of behaviors, the detection of repeated patterns, and measurements of social relationships and interactions. More accurate tracking is important, but accuracy alone is not enough. Researchers must also determine whether errors remaining after data preparation could change the particular behavior or social relationship being measured. A tracking method should therefore be considered suitable only when its remaining errors are sufficiently controlled for the intended behavioral conclusion.

## 1. Introduction

Quantitative measurement of animal behavior is a central goal in ethology, neuroscience, psychology, ecology, and animal welfare science. Earlier discussions of computational ethology emphasized both the promise of automated behavioral measurement and the limitations of manual scoring or highly constrained experimental designs [1,2]. Video recordings provide a rich, non-invasive source of behavioral information, and the recent development of deep learning-based computer vision has greatly changed how such data can be analyzed. Markerless pose estimation tools can achieve high spatial accuracy under evaluated conditions when annotations are reliable and the target species, viewpoints, and image conditions are adequately represented in the training and validation data [3,4,5], facilitating detailed analyses of posture, movement, and behavioral dynamics in freely moving animals. These advances have expanded the range of behaviors that can be quantified and have made it possible to study animals under less physically constrained conditions.

Despite these advances, multi-animal tracking remains substantially more difficult than single-animal pose estimation. In single-animal settings, the primary computational task is to localize body parts. In multi-animal settings, however, detected keypoints must also be grouped into animal instances and, when individual-level analysis is required, linked to persistent identities across frames [6]. This introduces additional problems of body-part assembly, temporal association, and identity maintenance. These problems become especially severe when animals are visually similar, densely clustered, or engaged in close social interactions. Occlusion and crowding can lead to missing keypoints, incorrect body-part assignment, and identity switches. As a result, the periods that are most important for understanding social behavior are often the periods in which tracking is least reliable.

Several deep learning-based tools and workflows now address different aspects of these challenges. In this review, multi-animal tracking is used as an umbrella term for methods that locate and follow multiple animals while maintaining the correspondence of observations to individuals over time. Depending on the method, the resulting representation may consist of identity-preserved centroid trajectories, full-body pose tracks, or both. Extensions of pose-estimation frameworks for multi-animal tracking, such as multi-animal DeepLabCut (maDLC) [7] and Social LEAP Estimates Animal Poses (SLEAP) [6], combine keypoint detection with strategies for grouping body parts and tracking individuals across frames. Other approaches, such as idtracker.ai, focus on maintaining individual identities from visual appearance and have demonstrated accurate trajectory tracking in large groups of unmarked animals [8]. More recent developments have reframed identity tracking as a representation learning problem, removing the requirement for video segments in which all animals are simultaneously visible [9]. Together, these approaches highlight that multi-animal tracking involves more than scaling single-animal pose estimation to multiple individuals. Instead, it requires multiple linked computational steps, including detection, pose estimation, identity assignment, temporal association, and error correction. Accurate tracking therefore remains difficult under occlusion and crowding, particularly during close social interactions. Workflow-level strategies such as vmTracking [10] have been developed to address these difficulties by improving pose and identity tracking under occlusion and crowding, thereby expanding the practical possibilities for quantitative social-behavior analysis.

For studies of social behavior, tracking accuracy alone is not sufficient. The resulting data must also support biologically meaningful analysis. Pose and trajectory data are increasingly used as inputs for downstream computational tools that classify predefined behaviors, identify recurring movement patterns, quantify social interactions, and relate behavior to neural activity. These approaches include supervised behavioral-classification tools, such as the Mouse Action Recognition System (MARS) [11] and Simple Behavioral Analysis (SimBA) [12]; unsupervised or semi-supervised methods for identifying recurring movement patterns, including Keypoint-MoSeq [13], B-SOiD [14], A-SOiD [15], and Variational Animal Motion Embedding (VAME) [16], together with the earlier Motion Sequencing (MoSeq) framework [17], which addressed the same goal using three-dimensional depth video rather than pose or trajectory data and which Keypoint-MoSeq [13] later extended to keypoint input; and analysis-oriented workflows, such as Deep Open Field (DeepOF) [18], that connect tracking outputs to behavioral analysis. Interaction-aware analyses are considered separately because they explicitly represent relationships among animals, whereas many classification and segmentation methods primarily characterize individual behavior. Recent reviews of computational mouse and rodent behavioral analysis have similarly emphasized the growing use of video-derived and tracking-derived data for automated behavioral phenotyping, particularly in disease-model studies and open-source rodent behavioral-analysis workflows [19,20]. However, the validity of these analyses depends on whether the tracking outputs are adequate for the intended behavioral inference, motivating the pipeline-level perspective developed in this review.

Several recent reviews overlap with individual components of this scope. These include surveys of deep learning-based multiple-animal tracking methods, datasets, and evaluation metrics [21]; pose-based and non-pose-based approaches to animal behavior analysis [22]; integrated visual monitoring across detection, tracking, pose estimation, and behavior classification [23]; the development of animal pose tracking toward three-dimensional and multimodal neuro-behavioral applications [24]; and deep learning in animal behavior and ecological research [25]. A contemporaneous review by Chindemi et al. provides a methodological overview of the steps and tools used to study rodent social behavior, including animal tracking, feature extraction, classification, segmentation, validation, and interpretation [26]. The present review overlaps with these topics but places particular emphasis on how identity, pose, missing-data, and temporal errors in video-based multi-animal tracking relate to data preparation and downstream behavioral and interaction inference.

**Figure 1:**
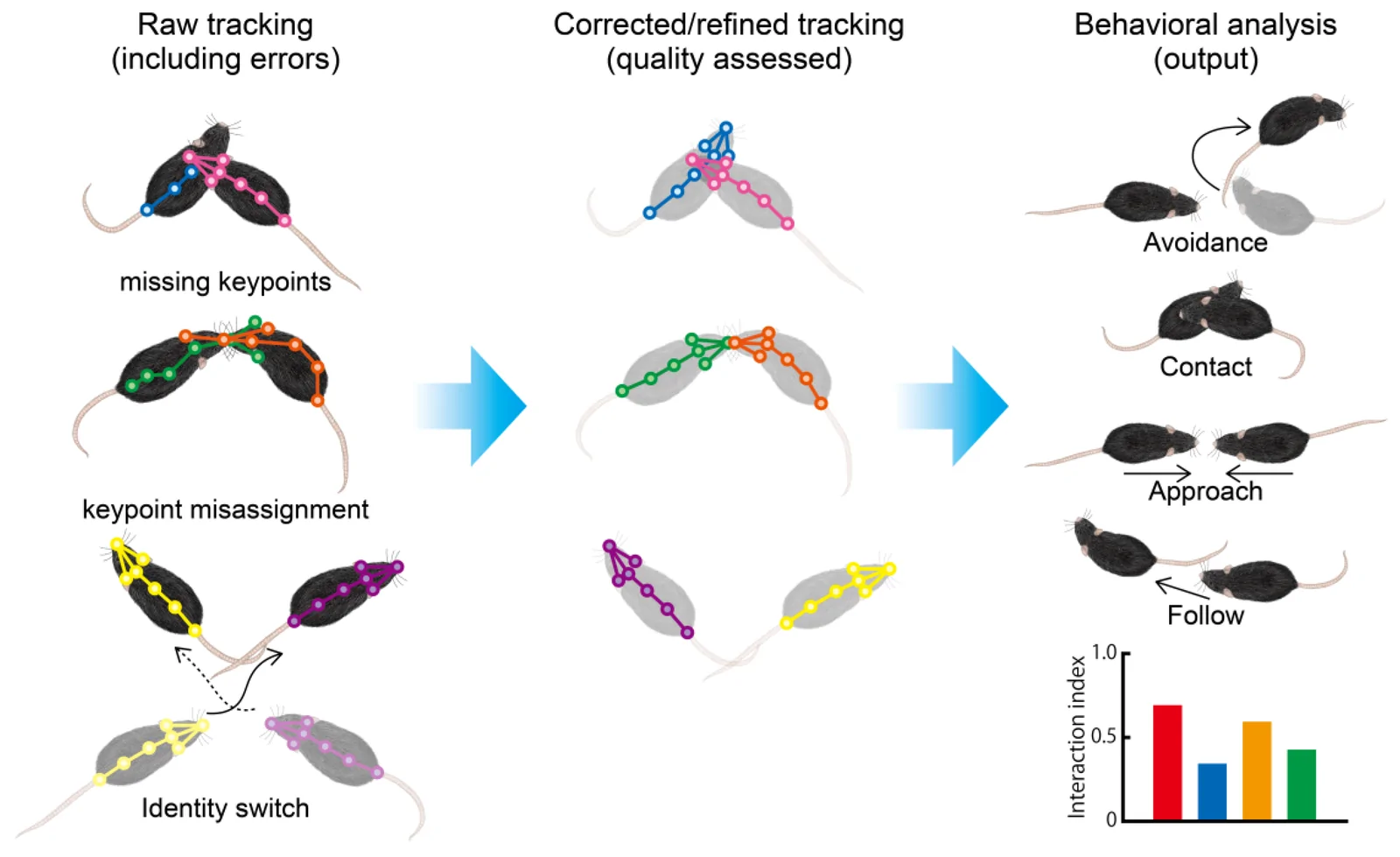
Preparing multi-animal tracking outputs for downstream behavioral analysis. Multi-animal tracking often produces raw outputs that contain ambiguities or errors, especially during close social interactions. Representative examples include missing keypoints, keypoint misassignment, and identity switches. Before downstream behavioral analysis, such outputs may require quality assessment, filtering, refinement, manual correction, interpolation or imputation, or exclusion according to the intended analysis. These procedures do not necessarily eliminate all residual errors or assumptions introduced during processing. Quality and provenance information—including confidence scores, missingness, interpolation or imputation status, and manual-correction history—is therefore carried forward with the tracking data from the quality-assessed stage to the downstream analysis shown at the right, rather than being discarded once the outputs have been refined.

This review discusses recent progress in deep learning-based multi-animal tracking and how tracking outputs can be connected to behavioral analysis. The main comparative synthesis focuses on tracking and tracking-derived behavioral analysis based primarily on 2D RGB video; systematic comparison of three-dimensional, multi-view, depth, and multimodal tracking systems is outside the scope of this review. The central perspective of this review is that multi-animal tracking should be evaluated in relation to the behavioral questions it is intended to support. Applying this principle requires workflows that make tracking quality and data provenance visible and validation procedures that are matched to downstream analyses. Figure 1 illustrates this central issue by showing raw tracking outputs that include representative errors, corrected or refined tracking outputs whose quality has been assessed, and their connection to downstream behavioral analysis. Depending on the intended analysis, these outputs may be handled through quality assessment, filtering, refinement, manual correction, interpolation or imputation, flagging, or exclusion, while retaining information about data quality and processing provenance.

Rather than treating tracking tools and behavioral analysis tools as separate topics, we therefore consider them as parts of a continuous workflow. We first summarize the major computational challenges in multi-animal tracking, including pose estimation, individual identification, occlusion handling, and long-term identity preservation. We then review representative tools and workflows, including identity-centered trajectory tracking, pose estimation-centered frameworks, human-in-the-loop refinement, and virtual marker-based approaches. Next, we examine how tracking outputs are prepared and used for downstream behavioral analysis, including feature-based social metrics, behavioral classification, behavioral segmentation, and interaction-aware analysis. Finally, we discuss validation, analysis-specific quality control, and future directions, emphasizing analysis that accounts for tracking quality and data provenance and the alignment of multi-animal tracking outputs with downstream behavioral interpretation. Box 1 defines terms that are used throughout the review and that may be unfamiliar to readers approaching multi-animal tracking from other fields.

Box 1 **Terms used throughout this review. These short definitions are intended for readers approaching multi-animal tracking from other fields; the corresponding sections give the fuller technical descriptions.**

**Pose estimation.** Estimating the positions of an animal’s body parts in each video frame, rather than only its overall location.**Keypoint.** A single tracked body part, such as the nose, an ear, or the tail base. A set of key-points describes an animal’s posture.**Identity switch.** An error in which the label identifying an animal is transferred to a different animal, so that the two trajectories are exchanged from that point on.**Occlusion.** A situation in which an animal, or part of it, is hidden from the camera, for example by another animal or by an object in the arena.**Ground truth.** Positions or behavioral labels annotated by people, used as the reference against which automated tracking or classification is assessed.**Missingness.** The absence of a tracked position for a body part or an animal in a given frame, whether because it was not visible or because the estimate was rejected during processing.**Interpolation and imputation.** Filling in missing positions by estimating them from surrounding frames. The resulting values are reconstructions rather than observations.**Provenance.** A record of how the tracking data reached their final state, including which values were measured, which were reconstructed, and which were corrected by hand.**Recurring movement patterns.** Short segments of movement that reappear across a recording. This review uses this plain expression for the general concept, because individual methods describe it in different ways.**Behavioral syllables.** The term used in some methods, notably MoSeq and Keypoint-MoSeq, for short recurring units into which a continuous stream of behavior is divided.

## 2. Methods

This article was prepared as a structured narrative review rather than a systematic review or meta-analysis. Literature identification combined database searches, citation tracking, and supplementary verification of relevant methodological papers and software information. Conceptual searches were conducted using combinations of terms related to multi-animal tracking, animal behavior, animal tracking, pose estimation, deep learning, artificial intelligence, occlusion, crowding, post-processing, behavioral classification, behavioral segmentation, and social interaction. Searches were performed in PubMed, bioRxiv, arXiv, Scopus, and IEEE Xplore. The search terms and the numbers of records identified, as of 15 September 2026, are summarized in Table 1.

Search strategies were adapted to the search functions of each database. PubMed, Scopus, and IEEE Xplore searches used the Boolean search expressions shown in Table 1. In PubMed, the expressions were entered directly into the standard search box without field tags and were processed according to PubMed’s default search mapping. bioRxiv searches used the Advanced Search interface with the ‘Full Text or Abstract or Title’ field and the ‘all’ option; because this interface does not support parentheses, each search theme containing alternative terms was implemented as a set of component searches, with the retrieved records accumulated in a single list whose size is the value reported in Table 1. arXiv searches used the Advanced Search interface with terms entered in the ‘All fields’ field, each group of alternative terms in one term box and the boxes combined with AND, with no subject category selected. In Scopus, the search scope was set to Article title, Abstract, and Keywords, and counts refer to the Documents results. In IEEE Xplore, the expressions were entered in the All Metadata field. No date, language, or document-type filters were applied in PubMed, bioRxiv, arXiv, or IEEE Xplore, and no date or language filters were applied in Scopus. The database-specific implementation of each logical search, the interface settings, and the retrieved-record counts are given in Table S1.

Database searches were supplemented by backward and forward citation tracking to identify related publications. Publisher pages were consulted to verify bibliographic information and publication status. Software repositories and official documentation were consulted to verify current, version-specific information on software availability and functionality. Software documentation was not used to support claims concerning methodological performance, generalization, or biological validity.

For the narrative synthesis, emphasis was placed on studies that were directly relevant to the methodological focus of the review, including original tool descriptions, methodological papers, protocols, broadly used workflows, and studies illustrating key practical or conceptual issues in multi-animal tracking and downstream behavioral analysis. Relevance was judged against the scope of the review, and a source was incorporated when it described a method, tool, or workflow for multi-animal tracking or for the analysis of tracking-derived behavior; documented a tracking, data-preparation, or validation problem affecting downstream behavioral inference; or provided the primary description of a method already discussed in the review. Studies focusing exclusively on human pose estimation, human action recognition, sports analytics, or human surveillance were not considered unless they introduced concepts directly relevant to animal-tracking methods; much of the underlying detection, pose-estimation, and multi-object-tracking methodology originates in that literature, which this review therefore does not cover comprehensively. Studies focusing only on single-animal pose estimation were considered when they provided methodological foundations for multi-animal tracking, downstream behavioral analysis, real-time pose estimation, or broadly pretrained animal pose models.

The review was developed as an iterative narrative synthesis, with database searches and citation tracking used to identify additional sources for topics requiring broader or more recent coverage. H.A. conducted the searches, assessed the relevance of identified sources, and decided which sources to incorporate into the narrative synthesis. The searches were used to map candidate literature and supplement the developing narrative rather than to define a predefined corpus for sequential title, abstract, and full-text screening; duplicate screening and reviewer-disagreement procedures were therefore not used. The use of single-author source selection is acknowledged as a limitation of the review. During revision, H.A. checked the correspondence between statements in the manuscript and the relevant passages of the cited publications and verified the bibliographic information of the cited references against publisher, PubMed, or official proceedings records. The resulting corrections and associated revisions to the manuscript were subsequently reviewed by both authors. The search records were combined and deduplicated after the searches had been completed in order to describe the size of the search output rather than to determine which sources were cited. Across the ten search themes and five databases, the counts shown in Table 1 sum to 3558 record occurrences. Because the same record could be returned under more than one theme or database, this total does not represent 3558 distinct records. After duplicates were removed within and across themes and databases by matching on DOI, arXiv identifier, or title where no identifier was available, the combined search output comprised 2209 unique records. Of the 51 sources cited in this review, 31 were present in the combined search records; the remaining 20 were identified through citation tracking, from the primary literature underlying the tools discussed, or from software documentation. This distribution reflects the design of the review, in which the searches were used to map and supplement a developing narrative, whereas much of the foundational and tool literature on which the synthesis depends was identified through the citation network of the field.

Across the full tracking-to-analysis pipeline, representative tools and workflows were organized in Table 2 into seven workflow categories according to their principal roles: identity-centered trajectory tracking, pose-estimation approaches, tracking refinement and correction strategies, integrated tracking-to-behavior pipelines and analysis workflows, behavioral classification methods, behavioral segmentation and recurring-pattern identification, and interaction-aware analysis. These categories were used to clarify the contribution of selected methods and were not intended to constitute mutually exclusive or exhaustive taxonomic classes. Table 2 provides illustrative examples representing distinct roles in the tracking-to-analysis workflow. It includes methods with demonstrated applications to multi-animal tracking as well as methods directly relevant to the analysis of tracking-derived animal behavior. The table is not intended to provide a comprehensive inventory, rank methods, or define field-wide coverage. Information used to classify and compare the tools was extracted by H.A. from the cited scholarly publications supporting each entry and checked against the relevant passages in those sources during revision. Publisher records were used to verify publication status and bibliographic information, whereas software repositories and official documentation were used only to verify current, version-specific information on software availability and functionality. Because this was a narrative review, no PRISMA flow diagram, meta-analysis, or pooled quantitative comparison was performed.

## 3. From Single-Animal Pose Estimation to Multi-Animal Tracking

Deep learning-based pose estimation has transformed video-based animal behavior analysis by enabling researchers to quantify body posture and movement without physical markers. Early markerless pose estimation frameworks, including DeepLabCut [3], LEAP Estimates Animal Pose (LEAP) [4], and DeepPoseKit [5], demonstrated that user-defined anatomical landmarks could be detected with high accuracy under the species, experimental settings, and recording conditions evaluated in those studies. These developments shifted behavioral analysis from coarse manual scoring toward high-dimensional, frame-by-frame quantification of posture and movement. In single-animal settings, the central computational problem is primarily keypoint localization: given an image or video frame, the model must estimate the position of predefined body parts. When the animal is clearly visible and well represented in the training data, this problem can often be solved with high accuracy.

The extension from single-animal pose estimation to multi-animal tracking introduces additional computational challenges. In multi-animal settings, detected keypoints must not only be localized, but also assigned to the correct body parts and individuals within each frame and linked to the same identities across time. Thus, multi-animal tracking requires the integration of keypoint detection, body-part assembly, identity assignment, and temporal identity tracking. These processes become especially difficult when animals are visually similar, interact closely, or occlude one another, leading to missing keypoints, incorrect body-part assignment, fragmented trajectories, and identity switches.

These requirements are addressed in major frameworks such as maDLC [7] and SLEAP [6]. Their designs incorporate mechanisms for body-part assembly, temporal association (including tracklet stitching in maDLC [7]), proofreading, and identity tracking, illustrating that multi-animal tracking requires more than a simple extension of single-animal keypoint detection. In other words, multi-animal tracking must preserve anatomical labels, individual identities, image positions, and temporal continuity at the same time.

Despite these advances, multi-animal tracking remains challenging in many situations that are relevant to social behavior analysis. Social interactions often involve close proximity, physical contact, following, chasing, investigation, aggression, or huddling, all of which can increase occlusion, crowding, and ambiguity in identity assignment. Thus, biologically informative moments may also be vulnerable to tracking errors. When identity, keypoint position, or temporal continuity is compromised during these events, downstream measures of distance, orientation, approach, avoidance, or posture may become biased or unavailable.

These considerations indicate that multi-animal tracking should be evaluated in relation to the intended downstream analysis. Some studies mainly require identity-preserved trajectories, whereas others require reliable anatomical keypoints, body orientation, and temporal continuity. This distinction motivates a pipeline-level view of multi-animal tracking, in which the purpose of tracking is not merely to produce coordinates but to provide measurements that can support behavioral and biological inference.

## 4. Representative Tools and Workflows for Multi-Animal Tracking

Deep learning-based multi-animal tracking has developed along several complementary directions. Some tools prioritize identity-preserved trajectories, large-group tracking, or re-identification, whereas others focus on estimating the full-body pose of multiple individuals or improving tracking reliability at the workflow level. These approaches should not be viewed as interchangeable solutions. Instead, they emphasize different components of the tracking pipeline, such as animal detection, identity assignment, trajectory linking, re-identification, body-part localization, animal assembly, and proofreading. In this section, we organize the tracking-related tools and workflows into four broad and partly overlapping categories: identity-centered trajectory trackers, pose estimation-centered frameworks, human-in-the-loop refinement and correction strategies, and integrated tracking-to-behavior pipelines and analysis workflows. The remaining workflow categories represented in Table 2 concern behavioral classification, behavioral segmentation and recurring-pattern identification, and interaction-aware analysis, which are discussed in later sections. Accordingly, the following comparison considers not only the outputs and workflow roles of representative approaches, but also the tracking or processing problems they address and the implications of their limitations for downstream behavioral analysis. Unless otherwise stated, performance descriptions in this section refer to evaluations reported in the original method publications. Such results characterize performance under the reported datasets and conditions and should not be interpreted as independent validation or evidence of general robustness across laboratories and experimental settings.

### 4.1. Identity-Centered Trajectory Tracking

One class of methods focuses primarily on preserving individual identities across time, often using centroid or body-level trajectories rather than full-body pose. These approaches are especially useful for collective motion, schooling, swarming, group coordination, and spatial organization, where the key requirement is to know where each individual is over time. In these contexts, detailed pose may be unnecessary, whereas identity continuity is essential.

idtracker.ai [8] is a representative identity-centered approach. It was developed to track all individuals in collectives of unmarked animals using visual appearance for identification. In the original framework, deep networks are used to detect when animals touch or cross and to identify individuals from images extracted from video segments. This differs conceptually from approaches that rely mainly on frame-to-frame continuity. Instead of simply linking nearby positions across frames, idtracker.ai attempts to recognize individuals by their visual features, thereby reducing the accumulation of identity errors after crossings or occlusions.

Recent developments in idtracker.ai further illustrate the shift from classification-based identity assignment toward representation learning. The new idtracker.ai [9] reframes multi-animal tracking as a self-supervised contrastive representation learning problem rather than a conventional classification problem. By learning an embedding space in which images cluster by animal identity, this approach is reported to eliminate the requirement for video segments in which all individuals are simultaneously visible, and to improve accuracy and substantially reduce tracking time [9]. The new framework therefore represents a broader trend in multi-animal tracking: identity preservation is increasingly being treated as a representation-learning problem rather than a purely temporal linking problem.

TRex [27] occupies a related but distinct position. It was developed as a fast multi-animal tracking system with markerless identification, particularly suitable for tracking large numbers of individuals using background subtraction and visual identification. Its strength lies in high-throughput trajectory tracking and identity preservation in large groups, making it particularly useful for studies of collective motion or group-level spatial organization.

A recent development in this direction is an unsupervised deep-learning method for multi-animal tracking (UDMT) [28] that uses transfer learning and does not require training annotations. UDMT combines bidirectional tracking, spatiotemporal transformer-based modeling, localization refinement, identity correction, and automatic parameter tuning. In the original study, the developers evaluated the method across multiple species and conditions including crowding, occlusion, rapid motion, and low image contrast [28]. These results characterize its performance under the datasets and conditions examined in that study, while robustness across independent laboratories and additional experimental settings requires separate evaluation.

Identity-centered trajectory trackers therefore provide an important foundation for multi-animal behavioral analysis. Their main contribution is to preserve individual identity and spatial trajectories over time, whereas pose estimation-centered frameworks add richer anatomical information when detailed posture, orientation, or body-part relationships are required. The appropriate representation depends on the downstream question and on whether the analysis requires identity-preserved centroid trajectories, full-body pose, or both.

### 4.2. Pose Estimation-Centered Frameworks

Pose estimation-centered frameworks aim to recover the positions of multiple body parts from multiple individuals while preserving the correspondence between anatomical landmarks and animal identities. These methods extend multi-animal tracking beyond identity-preserved trajectories by adding detailed anatomical information. They are particularly important when the scientific question depends on detailed posture, body orientation, contact, investigation, aggression, huddling, or other behaviors in which relative body-part positions are informative. In such cases, centroid trajectories alone are insufficient; the tracking output must preserve both anatomical structure and individual identity.

maDLC [7] and SLEAP [6] are representative tools in this category. maDLC extended the DeepLabCut framework [3] to multi-animal settings by combining keypoint localization with animal assembly, local tracking, global tracklet stitching, and identity prediction. SLEAP similarly provided a general-purpose framework for multi-animal pose tracking, with support for interactive labeling, training, inference, proofreading, top-down and bottom-up approaches, and identity tracking modules [6]. Both frameworks illustrate that multi-animal pose tracking is not simply a matter of detecting more keypoints; detected body parts must also be grouped into animal instances and associated with stable identities over time.

A useful distinction in multi-animal pose estimation is between bottom-up, top-down, and hybrid strategies. Bottom-up approaches first detect body parts across the image and then group them into individuals, which can lead to ambiguity when animals overlap. Top-down approaches instead localize each animal before estimating its pose and therefore depend on reliable instance detection, which becomes difficult when animals are close together or partially occluded. For example, bottom-up conditioned top-down pose estimation uses a bottom-up pose model in place of a generic object detector, so that the bottom-up stage supplies not only a bounding box but also a pose proposal that is passed as a condition to an attention-based top-down model [29]; recent versions of DeepLabCut support top-down, bottom-up, and hybrid approaches, including this approach [29,30].

Rather than ranking maDLC and SLEAP universally, this review focuses on how their outputs can be prepared, evaluated, and interpreted for downstream behavioral analysis. Both frameworks aim to provide multi-animal pose information while preserving anatomical labels, individual identities, and temporal continuity across frames. However, because they depend on correct body-part assembly and identity assignment, their performance can still be challenged by visually similar animals, close contact, occlusion, and crowding.

### 4.3. Human-in-the-Loop Refinement and Correction Strategies

A third category consists of strategies that improve tracking reliability by modifying the workflow rather than replacing the underlying tracking architecture. These strategies are particularly relevant for difficult frames in which fully automated tracking is uncertain or unstable. In such cases, targeted human intervention, iterative refinement, or workflow-level modification can substantially improve the quality of the final dataset.

In this context, human-in-the-loop tracking mainly refers to iterative human correction and model refinement. Users label representative training frames, train models, inspect predictions, correct difficult frames, retrain models, and proofread final outputs. SLEAP explicitly supports this iterative process by allowing predictions to be inspected and corrected, thereby enabling users to improve models through targeted refinement [6]. Similar logic also applies to the DeepLabCut workflow, in which users can refine labels and retrain models to improve performance in difficult conditions [3,7]. A recent preprint describes the Deep learning-based Identity-Preserving Labeled-Object Multi-Animal Tracker (DIPLOMAT) [35], a multi-animal tracking package that operates on the per-frame body-part probability maps produced by bottom-up DeepLabCut or SLEAP models rather than on the point estimates that those tools would normally output. It applies a hidden Markov model trace across the video, constrained by estimated movement and inter-body-part distances and computed so that the traces of different individuals remain mutually independent, and it therefore addresses identity continuity without relying on visual differences between individuals. The probability maps are retained and reused in an editing interface, so that a correction made in one frame can be propagated by re-running the trace. In the reported evaluation, the automated stage alone reduced identity swaps relative to the corresponding stand-alone DeepLabCut and SLEAP pipelines on a benchmark of three-mouse videos whose ground truth had been annotated independently of all three pipelines [35]. DIPLOMAT therefore illustrates that workflow-level strategies need not consist only of manual correction: automated inference over retained probability information and an efficient correction interface can address the same bottleneck from two directions.

vmTracking [10], developed by the authors of the present review, is a workflow-based strategy that uses labels obtained from an initial multi-animal tracking stage as virtual markers. These markers are added to the video, which is then analyzed with a single-animal tracking method in which the keypoints of all individuals are treated as one keypoint set, since a single-animal framework cannot be configured for multiple individuals; identities are resolved from the virtual markers, while the animals themselves remain physically markerless. The approach uses the virtual markers as visible identity cues for subsequent pose tracking and is not tied to a single pose-estimation framework. In the original evaluation, vmTracking implementations based on DeepLabCut and SLEAP were compared with their conventional multi-animal counterparts using manually annotated ground truth in recordings of three or five mice that included frequent close contact, crowding, and partial visual occlusion between animals [10]. Improvements in matching, missing-detection, and identity-related measures were most consistently observed with the DeepLabCut-based implementation, whereas the SLEAP-based implementation also showed improvements in some of the evaluated comparisons; the reported gains were most pronounced in scenes with occlusion and crowding and were more limited in scenes without them [10]. Applications to fish and human dancers further demonstrated the feasibility of vmTracking under additional recording conditions. These findings characterize the evaluated vmTracking implementations and should not be interpreted as a general comparison of the underlying capabilities of DeepLabCut and SLEAP.

Because the virtual markers are generated from first-stage tracking results, persistent identity or keypoint errors in those results can be transferred to the marker-added video and act as misleading visual cues. The reported workflow therefore includes visual inspection of the first-stage outputs and correction of identity switches and deviations in the keypoints used as virtual markers before the marker-added video is generated [10,32]. The strategy is most applicable when the first-stage output provides sufficiently continuous identity cues and the remaining errors can be identified and corrected with a manageable level of proofreading. Its utility is limited when the first-stage output does not provide usable cues because an animal or the relevant body part is not visible, or when extensive missing detections prevent reliable construction of the virtual markers. vmTracking can therefore be regarded as an example of a human-in-the-loop workflow that combines automated tracking with targeted correction rather than as a general replacement for existing multi-animal tracking frameworks.

### 4.4. Integrated Tracking-to-Behavior Pipelines and Analysis Workflows

Some tools go beyond tracking itself and connect pose or trajectory outputs directly to behavioral classification or analysis. These tools are important because they demonstrate that tracking is often only one stage in a broader behavioral analysis pipeline. MARS [11] is a representative example. It was developed as an automated pipeline for pose estimation and behavior quantification in pairs of freely interacting mice, linking computer vision outputs to supervised classification of social behaviors. SIPEC [36] similarly integrates segmentation, identification, pose estimation, and behavioral classification for interacting primates and mice in complex environments. DeepOF [18] provides a complementary example by linking rodent motion-tracking data to supervised and unsupervised behavioral pattern recognition within an analysis-oriented workflow. Such tools are useful when the behaviors of interest are predefined or when tracking-derived movement data are used to identify behavioral patterns under the assumptions of the analytical workflow.

Recent work has also shown how tracking, segmentation, and clustering can be combined for automated analysis of social behaviors. The segmentation tracking and clustering system (STCS) reported by Tang and colleagues [37] provides a recent example of an integrated multi-animal tracking and social-behavior analysis pipeline. This type of work illustrates a current direction in which object segmentation, identity tracking, and behavioral clustering or classification are linked within a single workflow. Its role is best understood as an example of tracking-to-behavior integration rather than as a general replacement for pose-estimation or identity-tracking frameworks.

Integrated pipelines are valuable because they reduce the gap between tracking output and behavioral interpretation. However, reported accuracy is generally established under particular species, assays, camera views, annotation schemes, and recording conditions. For example, the pretrained MARS models were developed for a standardized resident-intruder assay, whereas application to other settings requires users to train pose and behavior models for the intended task [11]. SIPEC was evaluated in separate mouse and non-human-primate applications, but these evaluations do not demonstrate transfer of the same trained behavioral classifier across species or recording settings [36]. Performance measured using held-out data from the same experimental context should therefore be distinguished from external validation in a different laboratory, species, assay, or recording condition. In the absence of such external evaluation, within-context accuracy should not be interpreted as evidence of general transferability.

Detailed rodent-focused comparisons of commonly used tools, including pipeline coverage, hardware and software requirements, generalizability, documentation, and benchmarking, are provided by Chindemi et al. [26]. Rather than duplicating this tool-level inventory, the present review compares methods primarily according to the tracking representation they provide, their role in the tracking-to-analysis workflow, and their suitability for the intended downstream analysis. For the tools whose primary output is a tracking representation, Table S2 in the Supplementary Materials sets these properties out side by side, giving for each approach the representation produced, how identity is established, what the user must supply, the evaluation context reported in the cited source, and the analyses that the representation supports. Because the reported evaluation contexts differ between studies, Table S2 is intended to support the matching of a tracking representation to an intended analysis and not to rank the tools against one another. Identity-centered trajectory trackers are appropriate when individual locations and long-term trajectories are sufficient, including many studies of collective movement or group-level spatial organization. Pose-estimation frameworks provide anatomically detailed outputs for posture- and orientation-dependent analyses but generally require body-part annotation, model training, and additional processing to assemble poses and preserve identities during close interaction. Human-in-the-loop refinement adds inspection or correction steps, whereas integrated tracking-to-behavior pipelines can reduce the need to assemble separate analytical components but may be more closely tied to particular species, assays, camera views, or behavioral definitions. Computational requirements, processing speed, and practical group-size limits also depend on software version, model configuration, hardware, image resolution, and recording conditions and should therefore be evaluated for the intended dataset.

The integrated pipelines discussed above also differ in what the user must supply: MARS, SIPEC, and STCS carry out tracking or segmentation within the workflow itself, whereas DeepOF operates on tracking output generated elsewhere. In many projects, users instead build modular workflows: pose or trajectory data are generated using tools such as maDLC, SLEAP, idtracker.ai, TRex, or vmTracking, and then processed by downstream analysis packages or custom scripts. This modular approach is flexible, but it places greater responsibility on the user to check tracking quality, define behavioral features, document preprocessing, and ensure that the tracking output is appropriate for the intended analysis. The following sections therefore shift from the tools themselves to the persistent bottlenecks that affect multi-animal tracking and to the practical steps required to prepare tracking outputs for behavioral analysis.

## 5. Persistent Bottlenecks in Socially Relevant Tracking: Occlusion, Crowding, and Identity Preservation

Although multi-animal tracking tools have advanced rapidly, several bottlenecks remain persistent in socially relevant behavioral contexts. Among the most important are occlusion, crowding, and identity preservation. These problems are closely linked: occlusion reduces visible information, crowding increases ambiguity among neighboring individuals, and both conditions make it more difficult to assign detected body parts or trajectories to the correct individual. In social behavior studies, these challenges are especially consequential because close interaction is often not a nuisance condition but the behavior of interest [10].

Occlusion occurs when one animal partially or completely blocks another animal, or when body parts are hidden by the animal’s own posture, another individual, or objects in the environment. Crowding occurs when multiple individuals are spatially close enough that body boundaries, keypoints, or trajectories become difficult to separate. In such scenes, tracking errors may include missed detections, false detections, incorrect body-part assembly, fragmented trajectories, and identity switches [6,7,10]. These errors can affect different aspects of the tracking output. A missing head or nose keypoint can make investigation or orientation measures unavailable or unreliable, an incorrect body-part assignment can distort estimated posture, and a broken trajectory can increase the likelihood of identity errors during subsequent linking or re-identification.

Identity preservation becomes particularly difficult when animals are visually similar. In many laboratory studies, animals may share the same strain, coat color, body size, or external appearance. In such cases, appearance-based cues are weak, and identity may be maintained mainly through temporal continuity or learned visual representations. These strategies can be sufficient when animals remain separated, but they become ambiguous when animals contact, overlap, cross paths, or huddle. A short period of visual ambiguity can therefore have effects that extend beyond the occluded frames themselves, because an identity switch or fragmented trajectory during that period may cause subsequent frames to be linked to the wrong individual [8,9,27,28].

These bottlenecks directly affect behavioral interpretation because social measures depend on who did what to whom and when. An identity switch can reverse the apparent direction of approach, missing anterior keypoints can bias measures of investigation, contact, or orientation, and fragmented trajectories can distort estimates of following, chasing, or social proximity. Pose- and trajectory-based behavioral models likewise assume that the relevant identities, body parts, and temporal structure are preserved; otherwise, inferred movement patterns or behavioral labels may partly reflect tracking artifacts rather than behavior, especially in interaction analyses [10,11,13,18,37]; keypoint jitter, for example, has been shown to be mistaken for behavioral transitions by an unsupervised segmentation model [13].

The severity of these bottlenecks depends on the intended downstream analysis. Some analyses mainly require identity-preserved trajectories, whereas others require detailed body-part pose. For trajectory-centered analyses, errors in distal body parts may have little effect if the key variables are individual position, movement path, proximity, or group-level spatial organization. For analyses of fine-scale social behavior, however, stable identity and reliable pose information become more important. Measures of social investigation, attack, mounting, avoidance, or nose-to-nose contact may depend on specific anterior keypoints and body orientation. For neurobehavioral or physiological studies, identity errors can be particularly damaging because behavioral features must be aligned with individual-specific neural signals, stimulation events, or physiological measurements. Therefore, the required level of tracking reliability depends not only on the video quality or tracking algorithm, but also on the biological inference being attempted.

Beyond event-specific occlusion and crowding, tracking performance can deteriorate when deployment conditions differ from those represented in training and validation data. Changes in illumination, camera viewpoint, background, arena geometry, image resolution, frame rate, or animal appearance can produce domain shift affecting detection, pose estimation, and identity assignment. Heterogeneous recording environments may therefore cause performance to vary across recordings or sites in ways not captured by the original benchmark. Validation under the intended recording conditions, followed where necessary by task-specific annotation, retraining, or adaptation, is important before tracking outputs are used for behavioral inference.

This distinction leads to the next step in the tracking-to-analysis workflow: preparing tracking outputs for behavioral analysis. Once the major sources of error are recognized, the question is not only which tool performs best under benchmark conditions, but how the resulting coordinates, identities, and confidence information should be inspected, refined, documented, and interpreted for a specific analysis, as illustrated schematically in Figure 1.

## 6. Preparing Tracking Outputs for Behavioral Analysis

Tracking outputs are not automatically equivalent to analysis-ready behavioral data. As summarized in Figure 1, within-frame errors, such as missing or misassigned keypoints, and across-frame errors, such as identity switches, may need to be addressed before downstream analysis. Coordinates generated by tracking software often require inspection, filtering, transformation, refinement, or interpretation in relation to the behavioral question being addressed [28,33,34]. The issue is therefore not only whether tracking errors occur, but how uncertain, incomplete, or corrected tracking outputs are handled before behavioral variables are extracted.

### 6.1. Tracking-Quality Dimensions and Data Provenance

In this review, model-generated confidence, identity ambiguity, missingness, and data-processing provenance are treated as distinct forms of information. Keypoint or detection confidence is a model-generated score associated with a predicted location or detection. Raw likelihood or confidence scores are generally model-specific scores rather than calibrated probabilities that a keypoint or identity assignment is incorrect. A low model score should therefore be treated as a flag for evaluation rather than as a known error probability. Confidence thresholds should be validated against annotated or visually verified data for the intended task, and empirical calibration is required before the scores are interpreted probabilistically. Identity uncertainty concerns ambiguity in assigning observations to the same individual across frames, whereas missingness denotes absent or rejected observations. Interpolation or imputation status and manual-correction status are provenance labels indicating how a value entered the analysis rather than estimates of its correctness. Downstream classifier confidence is produced at a later analytical stage and should not be interpreted as a measure of tracking accuracy. Pose-estimation tools commonly provide keypoint or detection scores [3,6,7], and multi-animal tracking tools provide identity assignments or tracks [6,7,8,9,10], whereas explicit identity-ambiguity estimates and provenance labels for interpolation, imputation, or manual correction may require additional calculation or workflow-level recording [30,33,34]. The availability and meaning of these outputs vary across tools and software versions.

Performance metrics reported in method-development papers characterize different aspects of tracking quality under defined evaluation conditions. Keypoint-localization measures assess agreement between predicted and annotated body-part positions [3,4,5,6,7] and are usually reported either as a mean localization error or as the proportion of keypoints falling within a distance threshold, for example PCK- or OKS-type measures, whereas identity-switch counts and trajectory-based identification scores assess aspects of identity association and continuity across frames [6,7,8,9]; the latter are reported in various forms, including raw switch counts, percentage identification accuracy, and multi-object-tracking scores such as IDF1-, MOTA-, or HOTA-type measures, which are defined in the multi-object-tracking literature rather than in any single one of these studies. Model confidence provides additional output-level information but is not itself a direct measure of tracking error. These quantities are therefore not interchangeable: low keypoint-localization error does not establish persistent identity, and high identity-tracking performance does not establish accurate localization of behaviorally relevant body parts during close interaction. Reported values must also be interpreted in relation to the dataset, species, group size, frame rate, camera view, annotation protocol, and occlusion conditions under which they were obtained. Consequently, values from non-comparable benchmarks should not be used to rank tools. The specific measures used, and the way they are defined and thresholded, differ between studies, and the present review does not attempt to compare the measures themselves. However, benchmark performance does not by itself determine whether user-specific tracking outputs are suitable for a particular behavioral analysis. Published evaluations are usually conducted using defined datasets, annotation protocols, camera conditions, species, and behavioral contexts, whereas users often apply tracking tools to recordings that differ in animal appearance, group size, recording environment, behavioral repertoire, and frequency of occlusion. Thus, the practical relevance of tracking accuracy depends on both the data being analyzed and the behavioral variables to be extracted.

The relevance of these tracking-quality dimensions depends on the downstream analysis. Localization measures can indicate errors in body-part position but do not assess whether individual identities remain continuous across frames. Identity and trajectory measures can indicate association failures or track fragmentation but do not establish the anatomical accuracy of behaviorally relevant keypoints. Data completeness and temporal continuity form an additional practical dimension because missing or reconstructed samples can alter the duration, sequence, and timing of behavioral variables. Coarse measures of locomotor activity may tolerate small errors in distal keypoints if the body center or centroid remains stable. Analyses of social investigation, nose-to-nose contact, or head orientation require reliable tracking of anterior body parts. Measures of approach, avoidance, chasing, following, or dominance are especially sensitive to identity switches because errors in identity assignment can reverse the apparent direction of an interaction. More generally, pose- and trajectory-based downstream analyses can be affected by missing keypoints, noisy coordinates, false-positive labels, interpolation, or smoothing, because these operations change the time series from which behavioral variables are derived [33,34]. Interaction-rule inference from multi-animal trajectories [41,42] and Granger-causal or predictive-directionality analyses [40] both depend on the timing and directionality of movement relationships among individuals. For this reason, their interpretation can be affected by temporal discontinuities, identity errors, and gap-handling procedures.

### 6.2. Post-Processing and Validation of Reconstructed Data

Recent tools for post-tracking refinement and imputation illustrate how raw tracking outputs can be converted into more usable behavioral data. refineDLC [33] provides a post-processing pipeline for cleaning, likelihood- and displacement-based filtering, and interpolation of noisy DeepLabCut outputs. DISK [34] addresses missing skeleton data by learning dependencies among keypoints and their dynamics, and shows that imputation can increase the number of detected movement episodes and improve the robustness of statistical comparisons between experimental conditions. These examples highlight that filtering, interpolation, imputation, and refinement are not merely optional cleanup steps, but procedures that can shape the behavioral time series used for downstream analysis.

In many workflows, tracking outputs may be filtered using likelihood or confidence values, short gaps may be interpolated, coordinate traces may be smoothed, and abrupt jumps in position or body-part distance may be flagged as potential errors. Such procedures can improve the stability and usability of derived features, but they also introduce analysis-dependent assumptions. For example, linear interpolation has been reported to perform well on short gaps in sequences containing a large proportion of static or low-movement frames, and poorly on sequences composed entirely of actions; reconstruction error also increases with gap length and movement speed and varies across behavioral action classes [34]. Similarly, smoothing or refinement procedures may reduce high-frequency jitter and implausible jumps, but they must be selected so that brief behavioral events and biologically meaningful transitions are not obscured [33]. Thus, post-processing choices should be matched to the temporal scale and behavioral variables of interest.

Whether reconstructed values preserve the behavior of interest should be evaluated at the temporal and spatial scales relevant to the intended analysis. Depending on the analysis, validation may include sensitivity analyses across filtering thresholds and permitted gap lengths, artificial masking of visible observations to test reconstruction performance, and held-out comparisons with frames in which the relevant keypoints or identities can be verified. When reconstructed samples contribute materially to an analysis, observed, interpolated, and imputed samples and their respective proportions should be reported separately. Brief social events should not be inferred solely from reconstructed samples unless reconstruction has been validated at the temporal scale and for the behavior being analyzed.

### 6.3. Visual Inspection and Analysis-Specific Quality Control

Visual inspection and manual correction also remain relevant in many tracking workflows. Some errors are behaviorally important but computationally subtle. For example, identity switches or misplaced keypoints may not always produce obvious discontinuities in the coordinate traces, but they can still distort measures of investigation, orientation, or interaction direction. For this reason, automated error flags and visual review can play complementary roles. Full video review with tracking overlays is the most comprehensive form of quality control, but it may be impractical for long recordings or large datasets. In practice, many workflows may therefore combine automated flags with targeted review of behaviorally important segments, such as contact, chasing, huddling, attack, or approach–avoidance episodes. Selection for visual review should not rely exclusively on low-confidence or automatically flagged segments, because behaviorally consequential errors, including identity switches, may occur despite apparently high model confidence. Quality-control sampling should therefore combine flagged or low-confidence segments with randomly sampled, apparently high-confidence segments and should include the behaviors central to the biological hypothesis. When ambiguity remains high, flagging or excluding uncertain segments may be more appropriate than forcing a definitive correction.

Preparing multi-animal tracking outputs for analysis-specific use is inherently context dependent. The same gap, confidence drop, trajectory discontinuity, or ambiguous keypoint assignment may be negligible for one analysis but critical for another. Preserving these distinct forms of tracking-quality and provenance information can therefore be useful. Confidence values, missing-data flags, interpolation labels, proofreading status, and manual-correction flags may help identify tracking segments that require evaluation and assess whether derived behavioral variables depend on low-confidence, missing, reconstructed, or manually corrected data. For example, frames reconstructed by interpolation might be excluded from analyses of brief social contact but retained for coarse locomotor summaries. Similarly, behavioral classifiers or segmentation models can be evaluated with and without low-confidence or manually corrected periods when the conclusion may depend on these input characteristics.

An analysis-specific quality-control plan can define error categories in relation to the behavioral variables being extracted and specify how segments are selected for inspection. Sampling can include both ordinary recording periods and segments with frequent contact, overlap, rapid movement, or other conditions expected to challenge tracking. Studies should report the confidence thresholds and rules used to identify implausible coordinates, the maximum gap duration permitted for interpolation, smoothing methods and parameters, identity-correction procedures, and the proportions of frames or events inspected, corrected, excluded, interpolated, or imputed when these procedures affect the analyzed data. Independent checking of a subset may be useful where manual corrections alter keypoints or identities, and inter-rater agreement should be reported when manual judgments alter behavioral labels. The appropriate sampling fraction and checking procedure depend on the recording duration, expected error frequency, and intended downstream analysis.

### 6.4. Differential Measurement Error Across Experimental Conditions

The relationship between tracking quality and experimental conditions is another important consideration. Tracking performance can vary with animal appearance, background contrast, recording environment, and activity level [43]. Tracking errors may therefore differ between treatment groups, recording sessions, animals, or behavioral states, especially when movement speed, posture, visibility, or occlusion differs across conditions. If such differences are not examined, variation in tracking quality may complicate the interpretation of biological comparisons. Reporting dataset-level quality descriptors, such as missing-data rates, confidence distributions, identity corrections, interpolation rules, and the proportion of analyzed events that were manually inspected or corrected, may improve transparency, especially when these values differ across groups or conditions. Quality descriptors should therefore be examined separately for relevant experimental groups and behavioral states rather than only after pooling all recordings. Sensitivity analyses that exclude low-quality segments can indicate whether the biological conclusion depends on differential tracking quality. Reweighting may also be considered, but only when the relationship between the selected quality indicator and measurement error has been justified and validated.

Preparing tracking data for downstream behavioral analysis is therefore part of the inferential pipeline. In multi-animal studies, the most consequential tracking errors are often those that affect relational variables: which individual performed an action, toward whom it was directed, when it occurred, and how it changed over time. Existing tools and recent methodological developments point toward workflows that combine automated filtering, refinement or imputation, visual inspection, tracking-quality and provenance documentation, and transparent reporting. These steps provide the basis for the next stage of analysis, in which prepared pose or trajectory data are transformed into behavioral categories, recurring movement patterns, relational features, or interaction models.

## 7. From Tracking Outputs to Behavioral Classification and Segmentation

Once pose or trajectory data have been prepared for analysis, the next challenge is to transform coordinates into biologically interpretable behavioral variables. Tracking outputs are usually high-dimensional time series, in which each frame may contain the positions of multiple body parts, individuals, or centroids. These coordinates are rarely the final object of biological interpretation. Instead, researchers use them to extract behavioral categories, recurring movement patterns, postural dynamics, movement states, or social events. The choice of analytical method depends on whether the goal is to quantify predefined behaviors, discover behavioral structure, or characterize relationships among individuals. In this section, we therefore consider three broad and overlapping approaches: feature-based analysis, supervised behavioral classification, and unsupervised or semi-supervised behavioral segmentation.

### 7.1. Feature-Based Analysis and Supervised Behavioral Classification

Feature-based analysis is the most transparent approach because each variable has a direct spatial or temporal meaning. In multi-animal experiments, relational features such as inter-animal distance, relative orientation, head-to-body distance, and frame-to-frame change in inter-animal distance can provide quantitative descriptions of interaction [11,18]. The strength of this approach is interpretability, but its limitation is that it depends on the researcher’s assumptions about which features are behaviorally relevant. It may therefore miss behavioral structure that is not captured by predefined measures.

Supervised behavioral classification is useful when the behaviors of interest can be clearly defined and labeled. Tools such as MARS and SimBA classify user-defined behaviors from pose-derived features; MARS additionally incorporates image-derived (pixel-change) features [11,12]. Earlier interactive machine-learning approaches such as the Janelia Automatic Animal Behavior Annotator (JAABA) also established the value of using researcher-defined labels and tracking-derived features for automated behavioral annotation [38]. These methods can quantify specific actions such as attack, mounting, grooming, social investigation, chasing, freezing, or other experimentally relevant behaviors. In multi-animal studies, supervised classifiers can detect social events when the input features and training labels include the relevant social context, such as relative distance, orientation, or movement direction. Their main limitation is that the output is constrained by the behavioral definitions and examples used during annotation. Ambiguous behaviors, transitional states, context-dependent actions, and behaviors not represented in the training set may therefore be difficult to classify reliably. Evaluation should avoid frame-wise random splits when temporally adjacent frames from the same recording could occur in both the training and test sets; depending on the intended scope of generalization, separation at the video, session, pair, or individual level is more appropriate. Because behavioral classes may be imbalanced, class prevalence should be reported and frame-level metrics such as per-class precision, recall, F1 score, and confusion matrices should be interpreted accordingly. These measures should not be assumed to establish event-level accuracy; when event onset, duration, frequency, sequence, or temporal boundaries are the biological outcomes, corresponding event-level evaluation should be used. Claims extending beyond the training context should be supported by cross-session or independent validation [44].

### 7.2. Unsupervised and Semi-Supervised Behavioral Segmentation

Different methods and publications use terms such as movement motifs, behavioral syllables, or model-inferred states to describe related, but not necessarily equivalent, forms of recurring structure identified from continuous movement data. To avoid treating these method-dependent terms as interchangeable, this review generally uses the plain-language expression ‘recurring movement patterns’ when referring to the broader concept; method-specific terms are retained only when describing the outputs or formulations of the corresponding methods.

Unsupervised and semi-supervised segmentation methods address a different goal: identifying recurring movement patterns from movement dynamics without requiring predefined behavioral labels. MoSeq and Keypoint-MoSeq model behavior as sequences of short recurring units, often termed behavioral syllables in these frameworks, while B-SOiD and VAME similarly aim to discover structure in pose-derived time series [13,14,16,17]. A-SOiD extends this line of work by incorporating active learning and expert guidance, allowing users to refine behavioral classes and discover sub-actions with relatively limited labeled data [15]. These approaches can identify candidate recurring movement patterns that may not be apparent from manual scoring and can reduce complex pose trajectories into discrete or low-dimensional representations. The resulting outputs should not be assigned biological meaning solely on the basis of the states inferred by the model. These outputs can depend on the input representation, temporal scale, hyperparameters, model assumptions, and initialization; their stability should therefore be examined across repeated fits, across animals, sessions, and experimental conditions, and across relevant analytical settings; this is a methodological recommendation of the present review rather than a procedure reported in the cited studies. Comparisons across sessions require alignment of the inferred states, and ethological meaning should be supported independently rather than inferred from the model output alone.

For multi-animal studies, an important distinction must be made between methods that can be applied to multi-animal tracking data and methods that explicitly model interaction. Many pose-based segmentation methods are primarily designed to analyze individual behavioral structure. They can be applied separately to each animal in a multi-animal dataset, but this does not necessarily mean that they model social interaction. A method may identify locomotor, grooming, rearing, or investigation-like movement patterns in each animal while ignoring whether another animal is nearby, whether the behavior is directed toward a partner, or whether it occurs as part of a reciprocal exchange. To interpret such patterns socially, individual behavioral states often need to be combined with relational features such as distance, orientation, contact, relative movement, or role in an interaction.

Some integrated tools illustrate how tracking outputs can be used directly for behavioral classification or social-behavior analysis. Examples include MARS for supervised classification of mouse social behaviors [11], SIPEC for combined segmentation, identification, pose estimation, and behavior classification in interacting animals [36], DeepOF for supervised and unsupervised pattern recognition from rodent tracking data [18], and STCS for integrating segmentation, tracking, and clustering in social-behavior analysis [37]. In this context, the main point is that tracking outputs acquire behavioral meaning through the features, labels, clustering procedures, and assumptions used to define or discover behavior.

Temporal scale is also important when linking tracking outputs to behavioral interpretation. Some behaviors unfold over frames or fractions of a second, whereas others require repeated interactions over longer periods. Brief events such as attack initiation, escape, or nose-to-body contact require high temporal precision, whereas dominance, affiliation, or social preference may emerge only after aggregation across time. Effective temporal resolution is constrained not only by the video frame rate but also by preprocessing operations such as smoothing, interpolation, and temporal aggregation. A pipeline that is adequate for segmenting relatively sustained locomotor states may therefore fail to preserve the timing required to analyze rapid attack initiation or reciprocal social responses. Thus, behavioral classification and segmentation should be interpreted in relation to the temporal scale of the phenomenon being analyzed.

Overall, behavioral classification and segmentation methods greatly expand the value of tracking data by transforming coordinates into structured behavioral representations. For multi-animal research, the central issue is to connect individual pose dynamics, relational context, event structure, and biological interpretation. This leads naturally to interaction-aware analysis, where the focus shifts from describing what each animal is doing to estimating how individuals relate to and influence one another.

## 8. Interaction-Aware Analysis of Multi-Animal Trajectories

Interaction-aware analysis addresses a further level of behavioral interpretation: how individuals relate to and influence one another. Social behavior is inherently relational. It depends not only on the posture or movement of a focal animal, but also on the spatial, temporal, and directional relationships between animals. For this reason, multi-animal tracking data provide an opportunity to move beyond individual behavioral states toward quantitative analysis of interaction structure.

Such analyses address questions about approach, avoidance, following, chasing, leadership, reciprocity, and group-level organization. Does one animal approach or avoid another? Does the movement of one individual predict the future movement of another? Are interactions reciprocal or asymmetric? How do group-level patterns such as alignment, cohesion, dispersion, or leadership emerge from pairwise relationships? These questions require features or models that explicitly represent relationships among individuals.

### 8.1. Relational Features and Operational Definitions

The most direct approach is to compute relational features from pose or trajectory data. These include inter-animal distance, relative orientation, relative speed, approach and withdrawal velocity, time spent in proximity, body-part-specific distances, contact duration, and spatial role. Such features are interpretable and can be directly connected to behavioral definitions. When these features are derived from 2D video, they describe projected spatial relationships; apparent proximity or overlap does not necessarily establish three-dimensional distance or physical contact. For example, as illustrative operationalizations, social investigation may be defined as the nose of one animal being near the head or body of another while oriented toward it, whereas chasing may be defined by sustained movement of one animal toward another with a consistent directional relationship. Such definitions are not universal: distance, orientation, and velocity thresholds depend on species morphology, camera geometry, frame rate, and assay context. Operational definitions should therefore be validated against expert-scored behavior for the relevant species and assay, and sensitivity to plausible threshold choices should be reported when these thresholds determine behavioral events.

### 8.2. Social Networks and Collective Behavior

At larger scales, collective behavior and social-network approaches provide ways to characterize group structure from identity-preserved trajectories. Measures such as alignment, polarization, nearest-neighbor distance, cohesion, group density, spatial sorting, and interaction rules can describe how groups move and organize over time [41,42,45,46]. Network representations can further summarize individuals as nodes and interactions as edges weighted by proximity, contact, or association [39]. These approaches may not require full-body pose if identity-preserved centroid trajectories are available, but they depend strongly on temporal continuity and identity preservation. A brief identity switch may have limited effect on global group density, but it can distort estimates of following, leadership, individual-level interaction preferences, or inferred interaction rules. The resulting network also depends on how edges and observation windows are defined; robustness should therefore be examined across plausible edge definitions and temporal windows, and weighted networks are generally preferable to thresholded ones, because thresholding an association index or interaction rate has been found to produce high rates of both type I and type II errors and is not recommended other than for display; differences in observation effort should also be taken into account [39], as should the spatial constraints of the recording arena. Temporal networks can retain changes in relationships that may be obscured by aggregation, whereas null models matched to the sampling and network-construction process can be used to evaluate whether observed structure differs from an appropriate reference [39]. Proximity or association networks describe observed co-occurrence or spatial relationships; interpretation in terms of preference, dominance, leadership, or influence requires additional behavioral or directional evidence and is particularly sensitive to identity errors.

### 8.3. Predictive Directionality and Statistical Inference

More advanced approaches attempt to characterize directed or time-lagged predictive relationships from movement dynamics. The augmented behavioral model proposed by Fujii and colleagues provides an important example by combining Granger-causal/predictive-directionality analysis with theory-guided behavioral modeling of navigation and motion processes and applying this framework to trajectories from mice, flies, birds, and bats [40]. This approach illustrates how time-lagged predictive relationships can be interpreted in relation to modeled attraction, repulsion, and interaction rules. However, directional predictability is not identical to biological causation. Several properties of the data also affect how such relationships should be interpreted. Movement data are strongly correlated from one frame to the next, so successive frames do not provide independent evidence. The time lags examined must match the frame rate and the timescale over which animals are expected to respond to one another, since a lag range that is too short or too long can obscure a real relationship or suggest one that is not present. Many of these models further assume that the statistical properties of the movement data remain stable over the analyzed period, which may not hold when behavior changes across a session. Finally, the results are sensitive to preprocessing, because smoothing, interpolation, and the handling of gaps all alter the temporal structure on which the analysis depends. When analyses combine spatial measures across recording configurations or incorporate external data streams, spatial calibration and temporal synchronization should also be verified. Shared environmental cues, group-level constraints, unobserved variables, or common responses to external stimuli can produce apparent directional relationships; causal interpretation therefore requires assumptions and experimental evidence beyond temporal predictability.

Because interaction-aware analyses rely on relationships among individuals, they place strong demands on tracking data. Identity switches can reverse the apparent direction of an interaction, time-alignment errors can distort lagged relationships, and interpolation or smoothing can alter the temporal structure from which responses are inferred. Thus, the reliability of interaction-aware analysis depends not only on the analytical model, but also on how identity preservation, temporal continuity, and uncertain or reconstructed data are handled.

Multi-animal trajectory datasets may contain large numbers of frames and interaction events, but these observations are not necessarily independent biological replicates. Frames within the same recording are temporally dependent, and repeated interactions involving the same animals, dyads, groups, or sessions share biological and experimental sources of variation. Inferential analyses should therefore define the experimental unit according to the biological question and avoid treating frames, trajectories, or repeated events as independent samples when replication occurs at the level of animals, dyads, groups, or sessions. Where appropriate, mixed-effects or hierarchical models can be used to account for these dependencies.

The required tracking representation also depends on the interaction being analyzed. Some questions can be addressed using identity-preserved centroid trajectories, such as group cohesion, collective motion, broad attraction–repulsion dynamics, and interaction-rule inference [40,41,42,45,46]. Other questions require full-body pose, such as head-directed social investigation, body orientation during approach, defensive posture, mounting, attack posture, or nose-to-nose contact. Trajectory tracking and pose estimation should therefore be viewed as complementary sources of information rather than competing alternatives. The appropriate representation depends on the spatial and temporal scale of the social process being studied.

Overall, interaction-aware analysis extends multi-animal tracking beyond the description of individual behavior toward the quantitative study of relationships among animals. Relational features describe pairwise or group-level social structure, whereas interaction-rule and predictive-directionality approaches characterize asymmetric or time-lagged relationships among individuals. These approaches complement behavioral classification and segmentation by adding information about who interacts with whom, when, and in what direction. The central requirement is that tracking data preserve the identities, temporal structure, and spatial relationships necessary for the intended level of inference.

## 9. Future Directions: Toward Reliable and Interpretable Multi-Animal Behavioral Pipelines

Future development of multi-animal behavioral analysis should focus on three connected priorities: robust evaluation under challenging social conditions, analysis-aware treatment of tracking quality and data provenance, and validated extension to new models, recording settings, and applications.

First, identity preservation under occlusion, crowding, and close social interaction remains a central challenge because tracking can be compromised precisely during behaviorally informative contact, overlap, and rapid movement. Progress will require improved re-identification and ambiguity detection together with workflow-level refinement. Cross-laboratory benchmarking using shared datasets should complement evaluations on individual development datasets. Such benchmarks should include ground-truth identity and pose annotations during prolonged animal-animal occlusion and close contact, and should represent visually similar individuals, variable illumination, rapid movement, multiple species, and different group sizes, with identity switches annotated where they occur rather than consisting predominantly of segments in which identity is easily maintained.

Second, tracking-quality information and data provenance should be integrated with the behavioral inference being attempted. Confidence, missingness, interpolation or imputation, manual correction, and identity ambiguity have different meanings, and confidence thresholds require empirical validation or calibration when they are interpreted probabilistically. Reproducibility should therefore be considered across the tracking-to-analysis pipeline, with sufficient documentation of the data and training-set composition, model architectures and software versions, annotations, random seeds where relevant, preprocessing and correction procedures, operational behavioral definitions, and derivation of the final variables. Missing or misidentified body parts and identity errors propagate through the analysis pipeline and can substantially affect behavioral annotation [26]. The required emphasis depends on the analysis: head-directed investigation relies on anterior keypoints and orientation, whereas following, leadership, behavioral segmentation, and interaction-rule inference place different demands on identity continuity and temporal structure [39,40,41,42,45,46].

Third, emerging models and acquisition approaches should be evaluated according to their intended multi-animal application. DeepLabCut-Live! [47] and YORU [48] demonstrate low-latency pose feedback and frame-based behavior detection, respectively, but robust social closed-loop applications additionally require real-time identity continuity and validation under occlusion and crowding. Broadly pretrained models such as SuperAnimal may reduce task-specific annotation [31], although transfer still requires validation across species, viewpoints, image conditions, keypoint definitions, and behavioral variables. Three-dimensional, multi-view, depth, and multimodal approaches may better represent interaction geometry, but their use also depends on acquisition, calibration, synchronization, and identity association under social conditions.

Extension beyond controlled laboratory settings presents related validation challenges. The downstream literature considered here remains concentrated largely in rodent studies, whereas other taxa and less controlled environments introduce different sources of domain shift and tracking error. Video-based precision livestock farming provides one example in which illumination, occlusion, visually similar individuals, group recording, and management conditions can affect both identity tracking and the interpretation of behavioral variables [49,50,51]. Across these settings, progress depends on treating tracking as part of an inferential workflow and validating the final measurements against the biological or applied question.

## 10. Conclusions

Deep learning-based multi-animal tracking has expanded opportunities for the quantitative study of social and collective behavior. Its full value will be realized when pose and trajectory outputs can be converted into reliable, context-aware behavioral variables. Validation should therefore extend beyond generic tracking metrics to determine whether tracking errors are sufficiently controlled for the final biological variable and inference of interest. Reporting should document the recording and annotation context, dataset partitioning where applicable, pose and identity performance, the handling of missing or processed data, manual correction, and outcome-specific validation. By aligning tracking quality and data provenance with downstream behavioral and interaction analyses, the field can better characterize the dynamic structure of animal social behavior and support stronger tests of hypotheses about its underlying biological mechanisms.

## Figures and Tables

**Table 1:** Database search terms and records identified as of 15 September 2026.

Search Theme	Search String	PubMed	bioRxiv	arXiv	Scopus	IEEE Xplore
Multi-animal tracking	(“multi-animal” OR “multi animal”) AND tracking	34	214	14	46	4
Multi-animal behavior	(“multi-animal” OR “multi animal”) AND behavior	48	416	16	63	5
AI/deep learning-based animal tracking	“animal tracking” AND (AI OR “deep learning”)	28	95	3	74	58
Animal pose estimation	animal AND “pose estimation” AND (AI OR “deep learning”)	113	44	26	213	90
AI/deep learning-based animal behavioral analysis	animal AND (“behavior analysis” OR “behavioral analysis”) AND (AI OR “deep learning”)	193	117	10	287	89
Occlusion, crowding, and overlap in animal tracking	“animal tracking” AND (occlusion OR crowding OR overlap)	27	37	5	53	25
Social behavior and interaction in animal tracking	“animal tracking” AND (“social behavior” OR “social interaction”)	20	163	2	47	2
Post-processing of animal pose-estimation outputs	animal AND “pose estimation” AND (post-processing OR refinement OR imputation)	20	4	15	22	10
Animal behavior classification and recognition	“animal behavior” AND (classification OR recognition) AND (AI OR “deep learning”)	129	65	15	353	112
Animal behavior segmentation and motif analysis	“animal behavior” AND (segmentation OR motif) AND (AI OR “deep learning”)	37	17	3	59	16

Search counts were used to document the scope of the narrative search and were not used as quantitative evidence of topic importance. The counts displayed across the ten search themes and five databases sum to 3558 record occurrences. Because the same record may appear under more than one search theme or in more than one database, this total does not represent 3558 distinct records or a deduplicated screening population. Scopus counts refer to records displayed in the Documents results.

**Table 2:** Representative methods, tools, and workflows in the tracking-to-analysis pipeline.

Workflow Category	Representative Tools or Methods	Main Output	Main Role in the Workflow	Tracking or Processing Issue Addressed	Implication for Downstream Behavioral Analysis	Representative Species and Settings
Identity-centered trajectory tracking	idtracker.ai ([8], JA), new idtracker.ai ([9], JA), TRex ([27], JA), UDMT ([28], JA)	Identity-preserved trajectories	Longitudinal identity tracking across animals	Long-term identity continuity and re-identification	Identity errors compromise individual trajectories and interaction histories; these systems provide positions and, in the case of TRex, outline- and midline-based posture with head–tail orientation, rather than multi-keypoint anatomical pose.	Fish, insects, rodents, and nematodes; controlled laboratory recordings
Pose-estimation approaches	DeepLabCut ([3], JA) (with a bottom-up conditioned top-down pose-estimation approach ([29], CP) supported in current versions ([30], DOC)), maDLC ([7], JA), SLEAP ([6], JA), LEAP ([4], JA), DeepPoseKit ([5], JA), SuperAnimal pretrained models ([31], JA)	Keypoints, skeletons, pose tracks	Markerless pose estimation and multi-animal pose tracking	Keypoint localization (single-animal frameworks [4,5]), body-part assembly and multi-animal pose tracking (multi-animal frameworks)	Missing or incorrectly assembled keypoints and identity switches can distort posture- and interaction-based measures.	Rodents, fish, insects, non-human primates, and other experimental species; mainly laboratory recordings, with some field-derived and in-the-wild data (drone-recorded zebras [5]; in-the-wild quadruped images [31])
Tracking refinement and correction strategies	vmTracking ([10], JA) ([32], PA), proofreading or correction workflows ([6], JA) ([7], JA) ([30], DOC), automated post-processing and imputation tools ([33], JA) ([34], JA), DIPLOMAT ([35], PR)	Refined, corrected, or imputed pose and identity-preserved outputs	Improvement of tracking reliability through human-guided or automated refinement, correction, and imputation	Occlusion, crowding, identity errors, noisy keypoints, and missing tracking data	Refinement can improve continuity and completeness, but propagated errors and correction or imputation procedures can alter downstream time series.	Rodents in controlled laboratory recordings for vmTracking, DIPLOMAT and the proofreading workflows; refineDLC was evaluated in dairy cattle and in field-recorded horses, and DISK across mouse, rat, fish, fly and human skeletons
Integrated tracking-to-behavior pipelines and analysis workflows	MARS ([11], JA), SIPEC ([36], JA), STCS ([37], JA), DeepOF ([18], JA)	Behavior labels, clusters, or behavioral patterns	Connecting tracking or pose outputs to behavioral analysis	Connection of tracking or pose outputs to behavioral analysis	Behavioral outputs depend on upstream tracking quality and validation across animals, sessions, and recording conditions.	Rodents and non-human primates, with additional tracking evaluation in ants and fruit flies [37]; controlled laboratory recordings, including social-behavior assays
Behavioral classification methods	JAABA ([38], JA), SimBA ([12], JA)	Behavior labels or classifiers	Supervised or semi-supervised behavior annotation/classification	Classification of predefined behaviors from tracking-derived features	Tracking errors, feature definitions, class imbalance, and validation design affect classification results.	Rodents and flies; controlled laboratory recordings
Behavioral segmentation and recurring-pattern identification	MoSeq ([17], JA), Keypoint-MoSeq ([13], JA), B-SOiD ([14], JA), A-SOiD ([15], JA), VAME ([16], JA)	Recurring movement patterns or model-inferred states	Unsupervised, self-supervised, or active-learning-assisted identification of recurring movement patterns	Identification of recurring movement patterns or model-inferred states	Tracking noise, preprocessing, temporal scale, representation, and model parameters can change the identified states.	Primarily rodents, with reported applications to fruit flies [13,14], non-human primate pose data [15], and human pose data [14]; controlled laboratory recordings
Interaction-aware analysis	Social network analysis ([39], JA), Granger-causal/predictive-directionality trajectory analysis ([40], CP), augmented behavioral models ([40], CP)	Interaction metrics, directional relations, social structure	Interpreting relationships among individuals	Construction of relational, network, or directional measures from trajectories	Interpretation depends on identity continuity, temporal resolution, preprocessing, edge definitions, and confounding; directional predictability does not establish biological causation.	Mice, flies, birds, and bats; laboratory and field-derived trajectory datasets

Note: Evidence-source abbreviations: JA, peer-reviewed journal article; PA, peer-reviewed protocol article; CP, peer-reviewed conference paper; PR, preprint; and DOC, official software documentation. Species and settings indicate representative examples from the cited sources and are not intended as exhaustive validation ranges for each tool or method. Tool and method abbreviations: UDMT, unsupervised deep-learning method for multi-animal tracking; maDLC, multi-animal DeepLabCut; SLEAP, Social LEAP Estimates Animal Poses; LEAP, LEAP Estimates Animal Pose; DIPLOMAT, Deep learning-based Identity-Preserving Labeled-Object Multi-Animal Tracker; MARS, Mouse Action Recognition System; STCS, segmentation tracking and clustering system; DeepOF, Deep Open Field; JAABA, Janelia Automatic Animal Behavior Annotator; SimBA, Simple Behavioral Analysis; MoSeq, Motion Sequencing; and VAME, Variational Animal Motion Embedding.

## Data Availability

No new data were generated or analyzed in this study. Data sharing is not applicable.

## Supplementary Materials

The following supporting information can be downloaded at https://www.mdpi.com/article/10.3390/ani16203191/s1, Table S1, search log for the database searches summarized in Table 1, giving for each search theme and database the database-specific implementation of the logical search, including the component searches used for bioRxiv and the individual term boxes used for arXiv, together with the searched fields, interface settings, applied filters, search date, and number of records retrieved; and Table S2, a comparison of the tracking representations produced by the tools and workflows discussed in Section 4.1, Section 4.2 and Section 4.3, giving for each the representation produced, how identity is established, what the user must supply, the reported evaluation context, and the analyses the representation supports.
